# Perceptions of Healthcare Safety Nets among Tertiary Hospital and Long-Term Care Hospital Nurses during the COVID-19 Pandemic: A Q-Methodological Approach

**DOI:** 10.3390/healthcare11202732

**Published:** 2023-10-13

**Authors:** Bom-Mi Park, Mi Young Chon, Hyun-Jung Lee

**Affiliations:** 1 Department of Nursing, Research Institute for Biomedical & Health Science, Konkuk University, Chungju-si 27478, Republic of Korea; spring0317@kku.ac.kr; 2 Department of Nursing, Seoul ST. Mary’s Hospital, The Catholic University of Korea, Seoul 06591, Republic of Korea; leehj8505@korea.ac.kr

**Keywords:** perception, healthcare safety net, nurses, COVID-19 pandemic, Q-methodological

## Abstract

This study was conducted to identify the types of perceptions toward healthcare safety nets. This study applied a Q-methodology. From a Q-population of 91 samples that included a review of the related literature and interviews with five tertiary hospital nurses and five long-term care (LTC) hospital nurses, 33 Q-samples were selected. The data were analyzed with the PC-QUANL program. We recruited 32 nurses in a tertiary hospital and 33 nurses in an LTC hospital. The perceptions of the healthcare safety net of tertiary hospital nurses were categorized into four types: (1) systematic system request; (2) realistic work support; (3) government support; and (4) emotional support. The perceived subjectivity of the healthcare safety net of LTC hospital nurses were categorized into four types: (1) reward system and facility environmental support; (2) realistic work support; (3) social prevention infrastructure support; and (4) government support. This study provides basic data for these different hospital settings, as well as to inform future government policy and system improvements in an era characterized by infectious diseases. Specifically, this study presents the types of perceptions of healthcare safety nets of nurses in two hospital settings that deliver care for patients.

## 1. Introduction

### 1.1. Background

As of 21 September 2023, the number of COVID-19 confirmed cases was 770,778,396 and the number of deaths was 6,955,141, and these numbers continue to increase [1]. The increase in COVID-19 cases strained the already-overloaded healthcare systems of various countries worldwide [2]. A healthcare safety net encompasses the securing of sufficient healthcare capacities, health equalities, accessibilities, and education, as well as the appropriate provision of healthcare services, such as medical insurance, protective equipment, medicines, examination, and medical services for all [3]. And safety-net hospitals provide essential care to people regardless of their financial circumstances and coverage insurance [4]. Public health promotion requires the existence of a healthcare system. In particular, to prevent disease outbreaks, it is important for standard operating procedures for infection control to be in place and for healthcare personnel to be aware of ways to prevent nosocomial infections. Nurses constitute the largest group of medical professionals, so when faced with the possibility of large-scale infections, medical institutions need to rely on nursing staff for services, which entails protecting nursing staff, cooperating with the government, and establishing a cooperative quarantine system to combat related diseases [5].

During an infectious disease epidemic, tertiary hospitals provide specialized treatment for patients with severe cases, and this requires the appropriate coordination of human and material resources (e.g., bed adjustment and medical staff) [6]. Therefore, according to the Ministry of Health and Welfare (MOHW) of Korea, a tertiary hospital must meet the relevant requirements for human power, facilities, equipment, and patients; provide professional medical services for severely ill patients; and have negative pressure rooms [7]. Moreover, research shows that most LTC hospitals, which were described to be vulnerable to group infection during the beginning of the spread of COVID-19 [8], are aware of the risk of group infection in the institution, and of the seriousness of this potential situation, and should thus actively conduct infection control activities to enhance their infection control capabilities [9]. Regardless of such awareness, infection monitoring systems [10] and human power for infection control remain lacking in these institutions [11,12]. Fortunately, the MOHW of Korea has established infection prevention and management fees for LTC hospitals starting from 1 July 2023, with the aim of inducing the foundation of a permanent infection control system and efforts to improve the quality of infection prevention and management in Korea [13]. Still, the perceptions of nurses working in tertiary hospitals and of those working in LTC hospitals regarding these healthcare safety-net systems may differ because of environmental differences, such as the lack of such a system in the hospital and the institution’s workforce.

During the early stages of the COVID-19 pandemic, hospitals lacked personal protective equipment, COVID-19 patient screening and referral staff, resources, laboratories for testing, waiting space, infrastructure for dealing with other infection control problems, and other necessary human and material resources for patient treatment [14]. The prolonged nature of the pandemic has also caused nurses to experience work overload and high stress levels, increasing the demand for improved work environments and efficient nursing labor management in hospitals [15]. Furthermore, because nurses tend to prioritize patient care over own life, there is a lack of attention to their quality of life [16]. It remains that understanding the perceptions of nurses working in tertiary hospitals and LTC hospitals regarding healthcare safety nets, and finding effective ways to address related resource shortages, are important measures for securing the stability of the nursing workforce and the efficient management of medical work amid public health crises [17]. Some prior research that investigated the healthcare safety net focused on concept analysis [3], securing a safety net and protecting public health [18], rapid implementation of telepsychiatry [19], and informatics response [20]. Notwithstanding, there is a dearth of studies on the perception of nurses working in different hospital environments regarding healthcare safety nets.

The Q-methodology does not focus on explaining things from an outside perspective, but rather on understanding phenomena from an inside perspective. It is useful for studying the perceptions, attitudes, and values of specific groups, as well as their similarities and differences [21,22]. Therefore, this study employed the Q-methodology, which considers diverse opinions and experiences, to classify the perceptions of nurses working in different hospital settings. We chose tertiary hospitals and LTC hospitals because while the first treat many emergencies and seriously ill cases, the latter usually deliver care for patients with chronic geriatric diseases. Thus, these two hospital settings differ not only in the diseases and patients treated, but also in the healthcare systems and services provided. We hope that this study contributes to healthcare institutions and governmental policies by providing suggestions to improve hospital work environments and education on infectious disease response and prevention.

### 1.2. Study Aim

This study aims to categorize the perceptions of nurses in a tertiary hospital and an LTC hospital regarding the healthcare safety net. First, the perceptions of nurses in different hospitals regarding the healthcare safety net were typified. Second, the characteristics of each perception of the healthcare safety net of nurses in these two hospital settings were analyzed and described.

## 2. Methods

### 2.1. Study Design

This study employed the Q-methodology to explore the perceptions of the healthcare safety nets of nurses.

### 2.2. Selection of the Q-Population and Q-Sample

In this study, data were collected through literature reviews and in-depth interviews.

In-depth interviews were conducted with 5 nurses working in a tertiary hospital and 5 nurses working in an LTC hospital in S city from 1 July to 9 September 2022. Interviews lasted approximately 60 min and were conducted once per participant in a hospital conference room, or in a place preferred by the participant. During the interviews, data omission was minimized through audio-recording and handwriting notes about details of the interview. The interview questions were based on the relevant literature [3,18,23] on healthcare safety nets.

The following questions were included: “What do you think is the healthcare safety net?”; “How do you perceive the healthcare safety net in caring for patients?”; “What do you think are healthcare safety nets necessary while caring for COVID-19 patients?”; “Do you think there is a difference in the healthcare safety net perceived by nurses in tertiary and a LTC hospital? If so, what would it be?”; and “What do you think is necessary or needs improvement for the healthcare safety net to work properly?”. During the interview, the researcher focused on the participants’ stories and allowed them to talk comfortably. Important parts of the interview process were selected by the researchers and then shown to participants for them to confirm data accuracy, and additional questions were asked to participants if specific details about the topic at hand were required. After the interview, the researcher saved the participant’s random number, listened to the recorded content repeatedly for a short period, and endeavored to arrange the participant’s words as they were said.

In total, 91 Q-statements about the perceptions of nurses in a tertiary hospital and in an LTC hospital were extracted by referring to the in-depth interviews with the Q-population and the relevant literature [3,18,23]. The 91 Q-statements were categorized under the same subject, and similar statements or cases in which two contents were included in one statement were classified. In the current research, a final Q-sample of 33 was extracted. The extracted Q-statement was evaluated for validity by a nursing professor with extensive experience in Q-research, taken from five nurses in a tertiary hospital and five nurses in an LTC hospital.

### 2.3. Selection of the P-Sample

The P-sample refers to a sample that is forcibly distributed to ensure that the statements of the Q-sample are normally distributed in the Q-population, and it is recommended to use small samples [22]. Accordingly, with the permission of the participating tertiary hospital and LTC hospital located in S City, among the nurses who wished to participate in this study, 65 were sampled. The total sample included 32 nurses from a tertiary general hospital and 33 nurses from an LTC hospital, including 10 participants who participated in in-depth interviews.

### 2.4. Q-Sort

The Q-sample was classified from 8 September 2022 to 20 January 2023. The Q-sample classification process was conducted in a conference room, or a place preferred by the participant. Before Q-sample classification, participants in the P-sample received explanations about the card classification method. Q-classification was conducted according to the principle of distributing the statements selected as the Q-sample by participants [22]. The 33 Q-samples were read individually and arranged on a Q-distribution chart according to the most negative (−4), neutral (0), and most positive (+4) using a 9-point scale. After the classification, participants wrote about the reasons for choosing the two most positive and the two most negative statements.

## 3. Data Analysis

The collected data were scored from one to nine points (from the most negative to the most positive) in the Q-sample distribution. For the coded data, Q-type analysis was performed, and principal component type analysis was performed using PC-QUANL Program. Types were entered in various ways based on an eigenvalue of 1.0 or higher, and the type evaluated to be ideal was selected based on the calculated results.

## 4. Ethical Consideration

This study was approved by the C Hospital (KC22QIDI0469) and K University Institutional Review Board (7001355-202205-HR-549). Before data collection, participants received explanations about the study aims, the right to withdraw at any time, the non-use of recorded files for any purpose other than research, confidentiality, and privacy measures (e.g., participant information would be numbered), and they provided written informed consent. All participants were given a small gift.

## 5. Results

### 5.1. Formation of the Q-Types

#### 5.1.1. Tertiary Hospital Nurses

As a result of the type analysis with the P-sample as the axis, the perceptions of healthcare safety nets of nurses in a tertiary hospital were categorized into four types. In the type analysis, the four types explained 54.33% of the total variance. And the explanation was 33.29% for type I, 8.06% for type II, 7.15% for type III, and 5.83% for type IV. Among the thirty-two people in the P-sample, ten, seven, three, and five were classified into types 1, 2, 3, and 4, respectively (Table 1).

#### 5.1.2. LTC Hospital Nurses

As a result of the type analysis with the P-sample as the axis, the perceptions of healthcare safety nets of nurses in an LTC hospital were categorized into types. In the type analysis, the four types explained 55.42% of the total variance, and the explanation was 40.96% for type I, 5.7% for type II, 4.63% for type III, and 4.13% for type IV. Among the 33 nurses in the P-sample, 11, 10, 4, and 8 were classified into types 1, 2, 3, and 4, respectively (Table 2).

### 5.2. Characteristics of the P-Sample

#### 5.2.1. Tertiary Hospital Nurses

The 32 tertiary hospital nurse participants were all women with an average age of 31.2 years; 10 were married and 22 were unmarried. Among them, five had a master’s degree or higher, twenty-six had a college degree, and one had a junior college degree. Their total clinical and tertiary hospital experience was 7.6 years on average. Regarding whether the nurses considered themselves to provide adequate care, 25 answered “yes” and 6 answered “no”. They stated that twenty-three hospitals, five social, and three personal systems were difficult, and one person said that there was no difficult system (Table 1).

#### 5.2.2. LTC Hospital Nurses

Among the 33 LTC hospital nurse participants, thirty were women and three were men, with an average age of 46.6 years; 23 were married and 10 were single. Their total clinical experience was 14.5 years on average, and their LTC hospital experience was 7.2 years. Regarding whether the nurses considered themselves to provide adequate care, 28 answered “yes” and 5 answered “no”. A total of 28 hospitals and 5 social systems were found to be difficult (Table 2).

### 5.3. Characteristics by Type

#### 5.3.1. Tertiary Hospital Nurses

(1) Type I: Systematic System Request

Type I tertiary hospital nurses strongly agreed with Q29, Q4, Q11, and Q2 and strongly disagreed with Q15, Q18, and Q33 (Table 3). They showed a higher agreement with Q30, Q29, Q4, and Q21 compared to other types (Z diff ≥ +1.00) and a higher disagreement with Q20, Q3, and Q18 (Z diff ≤ −1.00; Appendix A).

Participant No. 14 (P14), with the highest type weight (2.62) among the type I nurses, was a 34-year-old married woman who was a university graduate. Her total clinical experience and length of service at a tertiary hospital were 11 years and 7 months, respectively. When asked if she thought she had nursed adequately, said “no”, and when asked about a difficult situation, she said the hospital system. She agreed the most with Q4 (“I think it is necessary to ensure measures to protect from infection and for early detection, such as testing, self-isolation, hand washing, and wearing a mask”) and disagreed the most with Q15 (“I think that even if I must work in a second shift because of a lack of human power, I can endure it because I have a sense of calling for the job”).

We examined the distribution of agreeing and disagreeing statements within the type, and the subjective contents when selecting the Q-statement of the P-sample participants. The examinations indicated that the perception of the healthcare safety net of type I tertiary hospital nurses was more focused (vs. the three other types) on “COVID-19 patients.” P14 said, “It is necessary to prepare manuals such as action guidelines and response scenarios for each situation in the case of an outbreak, of a disaster medical response, health education, and the establishment of a non-face-to-face treatment system. In addition, if a systematic manual is provided, emergency situations can be dealt with more quickly, and support from clinical trials and research systems is required.” She also said that “A sense of calling cannot be a fundamental solution, and I think that patients and medical staff should take precedence over healthy people in psychiatric treatment and counseling programs.” Therefore, type I nurses attached greater importance (vs. other types) to systems (e.g., systematic preventive education, research, and treatment systems), and this led it to being named “Systematic system request.”

(2) Type II: Realistic Work Support

Type II tertiary hospital nurses strongly agreed with Q8, Q10, and Q19 and strongly disagreed with Q15, Q24, Q23, and Q32 (Table 3). They showed higher agreement with Q22, Q10, and Q33 than the other types (Z diff ≥ +1.00) and higher disagreement with Q7, Q2, and Q23 (Z diff ≤ −1.00; Appendix A).

Participant No. 30 (P30), with the highest type weight (2.11) amongst the type II nurses, was a 28-year-old single woman who was a university graduate. Her total clinical experience and length of service at a tertiary hospital were five years and six months, respectively. When asked if she thought she had nursed adequately, she said “yes”, and when asked about a difficult situation, she said the hospital system. The reason she agreed the most with Q10 (“I think it is needs to secure the number of nurses when nursing patients with infectious diseases”) was that “Medical workers are more motivated and responsible for their work when they feel that their work is being treated fairly, as well as when they receive financial compensation for it.” She disagreed the most with Q15 (“I think that even if I must work in a second shift because of a lack of human power, I can endure it because I have a sense of calling for the job”) as she thought that “it is important to motivate medical workers through appropriate compensation, rather than expecting them to persevere with a sense of calling for their job.”.

Again, we examined the distribution of agreeing and disagreeing statements within the type and the subjective contents when selecting the Q-statement of the P-sample participants. The perception of type II tertiary hospital nurses about the healthcare safety net was more focused (vs. the three other types) on the number of nurses to be secured. P30 described, “When nursing patients with infectious diseases, it is necessary to share information among medical personnel; in case of an emergency, it may be necessary to secure a helicopter or a security guard. There was no shortage of medical support, which should be used appropriately when necessary.” She also said, “To provide more support to people in medically vulnerable groups, differential services should be provided for the application of essential medical services regardless of medically vulnerable group.” Therefore, type II was named “Realistic work support” because these nurses placed greater importance (vs. other nurse types) on the direct work support necessary to perform nursing work in a hospital.

(3) Type III: Government Support

Type III tertiary hospital nurses strongly agreed with Q8, Q2, and Q7 and strongly disagreed with Q15, Q27, and Q28 (Table 3). They showed higher agreement with Q32, Q2, and Q3 than the other types (Z diff ≥ +1.00) and higher disagreement with Q28, Q29 and Q27 (Z diff ≤ −1.00; Appendix A).

Participant No. 15 (P15), with the highest type weight (2.69) among the type III nurses, was a 42-year-old married woman who was a university graduate. Her total clinical experience and length of service at a tertiary hospital were 19 years and 4 months, respectively. When asked if she thought she had nursed adequately, she said “yes”, and when asked about a difficult situation, she said the hospital system. She agreed the most with Q8 (“I think that an appropriate compensation system for overtime work should be established in the form of monetary compensation, vacations, awards, among others”) and described that, “It is regrettable that good talents are leaving to other places because compensation issues are not resolved.” She disagreed the most with Q15 (“I think that even if I must work in a second shift because of a lack of human power, I can endure it because I have a sense of calling for the job”) and said that “No one in the world wants to sacrifice themselves for work these days”.

Again, we examined the distribution of agreeing and disagreeing statements within the type and the subjective contents when selecting the Q-statement of the P-sample participants. The perception of type III tertiary nurses about the healthcare safety net was more focused (vs. the three other types) on care at the national and local government levels. P15 said, “In response and prevention and at the national level, the maintenance and support of medical personnel must be guaranteed, which requires sufficient basic personnel to ensure that the quality of services does not deteriorate.” She also reported, “I do not know if social distancing has a positive effect because it reflects many individual voluntary intentions, and there should be an infection support team. But I think more accurate manuals for specific work divisions and support are needed.” Therefore, type III was named “Government support” because nurses in this type were characterized by a focus on response, prevention, and education at the national and local government levels, as well as on support and compensation for the workforce.

(4) Type IV: Emotional Support

Type IV tertiary hospital nurses strongly agreed with Q28, Q8, and Q7 and strongly disagreed with Q14, Q22, and Q32 (Table 3). They showed higher agreement with Q28, Q18, and Q16 than the other types (Z diff ≥ +1.00), and higher disagreement with Q4, Q22, and Q11 (Z diff ≤ −1.00; Appendix A).

Participant No. 3 (P3), with the highest type weight (1.40) among the type IV nurses, was a 37-year-old married woman who was a university graduate. Her total clinical experience and length of service at a tertiary hospital were both 15 years. When asked if she thought she had nursed adequately, she said “yes”, and when asked about a difficult situation, she said the hospital system. She agreed the most with Q28 (“I think the infection support team should be able to provide quick support just like there is a CPR team”) and disagreed the most with Q22 (“I think it is necessary to secure sufficient transportation, such as helicopters and ambulances, in case of an emergency.”) because she thought the description in Q22 to be “applicable only to large hospitals”.

Again, we examined the distribution of agreeing and disagreeing statements within the type and the subjective contents when selecting the Q-statement of the P-sample participants. The perception of type IV tertiary hospital nurses regarding the healthcare safety net was focused more (vs. the three other types) on the following topics, which are represented by a quotation from P3: “infection support team, psychiatric treatment, and a counseling program was needed. An emotional nursing support system was also needed so that patients did not have anxiety about contracting COVID-19.” She also said, “Public awareness is needed for the common good rather than the individual, and it is necessary to prevent the spread of secondary infection by sharing information about it among medical personnel.” Therefore, type IV was named “Emotional support” because related nurses attached more importance (vs. the other types) to emotional support and an emotional support system for nurses.

(5) Consensus Viewpoints Among the Four Types

The four types coexist with each other and show independent characteristics. Nurses in all types agreed with Q9 (“I think there is a need for an isolation ward or facility where patients can be immediately isolated in the event of an outbreak”; z = 0.38) and they agreed the least with Q17 (“I believe that people who are not confirmed to have COVID-19 need emotional support from colleagues so that they can withstand the shifts”; z = −0.67) and Q26 (“I think it is necessary to rearrange the government organization that can operate a comprehensive and effective crisis communication system and a quarantine system”; z = −0.39; Appendix B). Tertiary hospital nurses thought that the need for isolation rooms and facilities that can isolate patients immediately upon occurrence was practically important, but they also acknowledged that the government was able to operate an effective crisis communication and quarantine system; thus, the importance of organizational reorganization was evaluated as low. In addition, although they sympathized with the need for psychiatric treatment and counseling programs, they did not agree with the need for emotional support from colleagues while carrying out heavy workloads.

#### 5.3.2. LTC Hospital Nurses

(1) Type I: Reward System and Facility Environmental Support

Type I LTC hospital nurses strongly agreed with Q8, Q4, and Q7 and strongly disagreed with Q15, Q3, and Q21 (Table 4). They showed higher agreement with Q20 and Q4 than the other Types (Z diff ≥ +1.00) and higher disagreement with Q3 (Z diff ≤ −1.00; Appendix C).

Participant No. 27 (P27), with the highest type weight (1.87) in type I, was a 30-year-old unmarried man who was a college graduate. The total clinical experience of nurses and the length of service in an LTC hospital were the same at two years and six months, respectively. When asked if he thought he had nursed adequately, he said “yes”, and when asked about a difficult situation, he said the hospital system. He agreed the most with Q8 (“I think that an appropriate compensation system for overtime work should be established in the form of monetary compensation, vacations, awards, among others”), and the reason was as follows (information within square brackets was added by the author for clarity): “If we are not compensated with money, awards, vacations, there is no reason to refer to overwork and overtime.” He disagreed the most with Q15 (“I think that even if I must work in a second shift because of a lack of human power, I can endure it because I have a sense of calling for the job”), and the reason was as described herein: “The quality of my personal life comes first and before my sense of calling.”.

We also examined the distribution of agreeing and disagreeing statements within the type, and the subjective contents when selecting the Q-statement of the P-sample participants. The perceptions of type I LTC hospital nurses about the healthcare safety net were more focused on topics that are exemplified by the following excerpt: “Money for overtime work is necessary to improve the practical compensation system such as vacations and prizes, as well as environmental improvements, such as protective equipment and resting areas.” However, psychological counseling and mental health management to reduce the anxiety, stress, and insomnia of medical personnel was not considered necessary as type I nurses deemed these issues to not be so high as to require treatment. Therefore, type I was named “Reward system and facility environmental support”, as nurses in this type attached more importance (vs. other Types) to direct material compensation and environmental improvements.

(2) Type II: Realistic Work Support

Type II nurses strongly agreed with Q8, Q10, Q28, Q9, and Q12 and strongly disagreed with Q15, Q24, Q23, Q25, and Q5 (Table 4). They showed higher agreement with Q28, Q3, and Q33 than the other types (Z diff ≥ +1.00) and higher disagreement with Q1 and Q4 (Z diff ≤ −1.00; Appendix C).

Participant No. 26 (P26), with the highest type weight (1.57) among the type II nurses, was a 56-year-old married woman who was a university graduate. Her total clinical experience and length of service in an LTC hospital were 4 years and 11 months, respectively. When asked if she thought she had nursed adequately, she said “yes”, and when asked about a difficult situation, she said the hospital system. She agreed the most with Q10 (“I think it is needs to secure the number of nurses when nursing patients with infectious diseases”), stating that “I believe fair compensation should be paid. I think the management of medical workers’ labor is too backward.” She disagreed the most with Q15 (“I think that even if I must work in a second shift because of a lack of human power, I can endure it because I have a sense of calling for the job”), and the reason for this was because “Maintaining the health status of medical personnel is also an important resource, and I believe that medical personnel have the right to be protected. I do not think that a system that only forces medical workers to sacrifice can be maintained for a long time.”.

Again, we examined the distribution of agreeing and disagreeing statements within the type, and the subjective contents when selecting the Q-statement of the P-sample participants. The perception of type II LTC hospital nurses about the healthcare safety net were more focused on (vs. the three other Types) securing an infection support team, supplementary personnel, nursing staff, and workforce. Therefore, they emphasize the need for support in nursing work. P26 said, “I do not think it is good for the country’s finances to provide essential care to all patients, and there is no immediate need for clinical research.” Therefore, type II nurses attach greater importance to direct work support (vs. other types) when performing nursing work in a hospital, so type II was named “Realistic work support”.

(3) Type III: Social Prevention Infrastructure Support

Type III nurses strongly agreed with Q9, Q12, Q11, and Q29 and strongly disagreed with Q33, Q20, and Q15 (Table 4). They showed higher agreement with Q24, Q6, and Q5 than the other types (Z diff ≥ +1.00) and higher disagreement with Q10, Q20, and Q33 (Z diff ≤ −1.00; Appendix C).

Participant No. 12 (P12), with the highest type weight (1.03) among the type III nurses, was a 62-year-old married woman who was a university graduate. The total clinical experience of the nurse was 20 years and the length of service in an LTC hospital was 8 years and 1 month. When asked if she thought she had nursed adequately, she said “yes” and when asked about a difficult situation, she said the social system. She agreed the most with Q9 (“I think there is a need for an isolation ward or facility where patients can be immediately isolated in the event of an outbreak”) and disagreed the most with Q20 (“I think we need a rest area where we can take a break while wearing protective gear”), and the reason for choosing Q20 was that, “I’m more concerned about adverse health effects that can come from overwork than with the provision of a resting place to stay in while wearing protective gear; therefore, shortening the shift time is more important.”

Again, we examined the distribution of agreeing and disagreeing statements within the type and the subjective contents when selecting the Q-statement of the P-sample participants. The perceptions of type III LTC hospital nurses about the healthcare safety net were more focused (vs. the other types) on “immediately isolating infected patients”. P12 said the following: “A one-step power source or transfer system is important in the case of infection and in the establishment of social infrastructure, education, and training for medical personnel. In addition, it is necessary to systematically respond to infected cases by establishing manuals, such as response scenarios for each situation.” She further described that “a fundamental solution is needed, rather than increasing the number of nurses or establishing a resting space in a hospital.” Therefore, type III LTC hospital nurses were characterized by attaching greater importance (vs. other types) to the establishment of social prevention support programs and systems that help prevent and preemptively deal with cases on the event of an infectious disease. Thus, this type was named “Social prevention infrastructure support”.

(4) Type IV: Government Support

Type IV nurses strongly agreed with Q10, Q8, Q4, Q9, Q1, and Q7 and strongly disagreed with Q24, Q25, and Q32 (Table 4). They showed higher agreement with Q1 than the other types (Z diff ≥ +1.00) and higher disagreement with Q28 and Q24 (Z diff ≤ −1.00; Appendix C).

Participant No. 33 (P33), who showed the highest type weight (1.30) among the type IV nurses, was a 55-year-old married woman who was a college graduate. Her total clinical experience and length of service in an LTC hospital were 18 and 14 years, respectively. When asked if she thought she had nursed adequately, she said “yes”, and when asked about a difficult situation, she said the hospital system. P33 agreed most with Q1 (“I think access to healthcare services should be guaranteed, such as using and receiving support from selected clinics”), and this was because she thought that “access to screening clinics is good, so that we can conduct a lot of tests and manage infected people.” She disagreed the most with Q28 (“I think the infection support team should be able to provide quick support to nurses, just like the CPR team”), and this was owed to the following: ““I think medical personnel support is necessary at the national level. And, it would have been better for us to support our hospital from the state rather than out-side support team.”

Again, we examined the distribution of agreeing and disagreeing statements within the type and the subjective contents when selecting the Q-statement of the P-sample participants. The perception of type IV LTC hospital nurses about the healthcare safety net was more focused (vs. other types) on “selective clinic operation and support”. P33 described the following on the topic: “The accessibility of medical services should be guaranteed so that infected people can be managed. In addition, disaster medical response and disease of infectious management measures at the national and local government levels must prevent the disease from spreading.” She further posited, “It would be nice to have an infection support team, but I think there are other ways to provide support now, and Korean society already has a fairly well-established digital infrastructure; therefore, the initial response and preparation of countermeasures are more important.” Therefore, type IV was named “Government support” because nurses in this type attached more importance (vs. other types) to securing nurses, guaranteeing general access to medical services, and supporting and compensating human power at the government and local government levels.

(5) Consensus Viewpoints Among the Four Types

The four types showed coexisting and independent characteristics. Nurses in all types (Z score ≥ +1.00) agreed with Q9 (“I think there is a need for an isolation ward or facility where patients can be immediately isolated in the event of an outbreak”), Q12 (“I think that a one-step power source and transfer system is needed in the event of infected patients”), and Q7 (“I believe that maintenance and support measures for stable healthcare personnel should be guaranteed at the national level”). Meanwhile, most nurses across types disagreed with Q15 (“I think that even if I must work in a second shift because of a lack of human power, I can endure it because I have a sense of calling for the job”; Z score ≤ −1.00; Appendix D). Nurses felt the need for quarantine facilities in hospitals so that they can be ready to deliver nursing care in a situation characterized by the rapid increase in patient numbers. In addition, since LTC hospitals tend to transfer their patients to tertiary hospitals or infectious disease hospitals, nurses showed greater consensus about the importance of the transfer system. Additionally, they suggested that the ongoing human power shortage requires efforts to maintain and support the safety of medical staff at the national level. Still, when nurses were questioned about having to work a second shift because of a lack of human power, they reported that this was difficult to endure just through a sense of calling for the job.

### 5.4. Comparison of Q-Types between Tertiary Hospital and LTC Hospital Nurses

**Figure 1:**
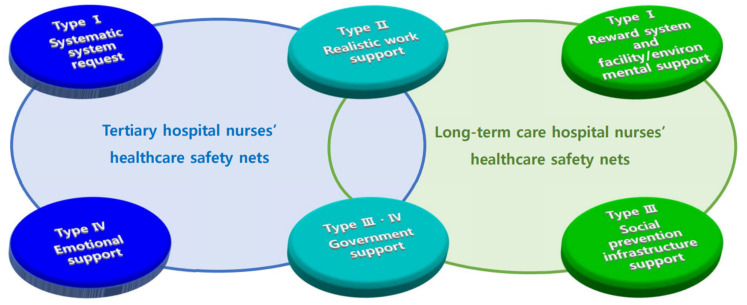
Perceptions of Healthcare Safety Nets Among Tertiary Hospital and LTC Hospital Nurses During the COVID-19 Pandemic.

In tertiary hospital nurses, type II (Realistic work support) and type III (Government support) were similar to LTC hospital nurses’ type II (Realistic work support) and type IV (Government support) categories. Meanwhile, the type I (Systematic system request) and type IV (Emotional support) categories of tertiary hospital nurses diverged from the type I (Reward system and facility environmental support) and type III (Social prevention infrastructure support) categories of LTC hospital nurses (Figure 1).

## 6. Discussion

In this study, the perceptions of nurses in a tertiary hospital and an LTC hospital regarding healthcare safety nets were categorized, the characteristics of each type were explored, and the similarities and differences between nurses working in each hospital were compared. The perception of tertiary hospital nurses about healthcare safety net was divided into four types (i.e., systematic system request, realistic work support, government support, and emotional support), and so was that of LTC hospital nurses (i.e., reward system and facility environmental support, realistic work support, social prevention infrastructure support, and government support). Moreover, the types II (realistic work support) and III (government support) in tertiary hospital nurses shared commonalities with types II (realistic work support) and IV (government support) in LTC hospital nurses. Thus, it seems that all hospital nurses perceived that they need realistic work support from the government to deal with the lack of resources and to tackle infectious disease outbreaks.

The type named “realistic work support” was the second recognition type in both tertiary hospital (21.9% were in this type) and long-term hospital care nurses (30.3% were in this type). This type refers to work support that is practical and complies with the real clinical needs of nurses, such as support for securing nursing workforce, sufficient emergency helicopters, and security personnel, as well as promoting information sharing among medical personnel. The appropriate provision of such support requires changes in infection control system management as there is the need to accommodate measures that help alleviate the shortages of personal protective equipment, supplies, and staff [24]. This is similar to the findings of prior research, which described the need for nursing fees or allowances [25]. Academicians have shown that a poor work environment and a lack of support affect burnout and turnover in medical personnel [26]. Therefore, the establishment of such a realistic work support system could have a positive impact on routine infectious diseases and on the outcomes of measures to deal with future epidemics.

The third and fourth recognition types for tertiary hospital (31.3%) and LTC hospital nurses (24.2%), respectively, were both named “government support”. This type referred to the response and prevention measures at the national and local government levels, as well as support for medical personnel at the national level. Governments and health systems should educate people on psychological and social cohesion, provide up-to-date information [27], and promote the prompt use of health information technology for public health responses to epidemic situations, such as the COVID-19 pandemic [20]. Researchers also show that providing training programs improve nurses’ abilities to care for patients and eventually improve patient care quality [28]. Although Korea was poorly prepared to deal with the pandemic, it recognized the threat early and made efforts to quickly activate national responses through multifaceted interventions [29]. Nevertheless, it remains necessary and important to anticipate an infectious disease outbreak, prepare relevant responses, and for stakeholders to have nursing administrative skills that help minimize the impact of sudden changes in human resource structures [30]. As the current and future eras may be related to more infectious diseases such as COVID-19, they may be marked by many situations that are difficult for individuals and hospitals to predict and resolve. This potential reality entails the need for continuous support from the government, which can help improve the healthcare safety net and eventually reduce morbidity and mortality rates.

The first recognition type in tertiary hospital nurses was the “systematic system request”, which corresponded to 31.3% of the total subjects. This type appeared in relation to remarks about the need for systematized manuals (e.g., on what to do when identifying COVID-19 patients and initial responses), for health education, for the establishment of a non-face-to-face treatment system, and for support for clinical trials and research. These findings are similar to those of previous studies on the use of healthcare safety net systems (e.g., health information technology systems) [20], the importance of vaccine development by researchers [31], and a telemedicine treatment system created because of a shortage of hospitals and medical staff [32]. These results emphasize that the government and hospitals should prepare systematic systems for dealing with infectious disease prevention, initial responses to outbreaks, and promoting clinical research on topics such as reinfection responses and disaster medical responses, management, and education.

The fourth recognition type among tertiary hospital nurses was “emotional support”, accounting for 15.6% of the participants. This type was based on the need for psychiatric treatment, counseling programs, and an emotional nursing support system. These characteristics of type IV are consistent with the findings of studies on nurses’ mental health and psychological experiences; these studies depict that nurses were experiencing high stress levels [2], and that anxiety, stress, and physical fatigue caused more anxiety and fear among nurses [2,33,34]. Healthcare workers have also been shown to be a stressed occupational group which are associated with negative worker outcomes [35], and exposure to COVID-19 in this group was reported to hinder one’s ability to protect loved ones from COVID-19 [36]. During the COVID-19 pandemic, there were many unexpected situations in which nurses struggled on the frontline of the healthcare system; they wore uncomfortable personal protective equipment, reportedly felt pushed to the healthcare “battlefield” without preparation, and had to care for patients [37] voluntarily while fearing that they could inadvertently spread the virus to people around them. Social activities were also restricted, and a Korean nurse in a past qualitative study reported the following, “I felt despair when my work was not properly recognized or when the end of the infectious disease was not in sight” [37]. There is a lot of demand for mental stress management among nurses, but related support and programs are still lacking in the field. For high-risk groups who need emotional support, it would be a good idea to provide support, such as continuous psychological counseling, in connection with a local mental health center. In particular, it appears that tertiary hospitals need to deploy psychological counselors to provide continuous emotional support to nurses.

The first recognition type among LTC hospital nurses was “reward system and facility environmental support”, accounting for 33.3% of the participants. This type was characterized by descriptions from nurses about the need for a practical compensation system (e.g., involving monetary rewards, vacation, and prizes for overtime work) and environmental improvements, including betterments regarding protective equipment and the provision of rest areas. Health systems have historically faced labor shortages and must thus further improve the awareness of governmental agencies regarding healthcare worker health, well-being, and retention [35]. In the recent situation of a prolonged pandemic, various problems and fragilities of healthcare systems came to the surface, such as a lack of beds, medical staff burnout, and workforce shortages. Regardless of the formation of this “vacuum” in the medical field, nurses reportedly remained, persevered, and developed a sense of calling and responsibility regarding their work. This does not reduce the importance of ensuring fair compensation for nurses, and the provision of appropriate quality and quantities of protective equipment [38]. Thus, the compensation system, facilities, and environmental support of nurses should be revamped at the hospital level.

The third type of recognition among LTC hospital nurses was “social prevention infrastructure support”, which accounted for 12.1% of the participants. This type describes the need for systematic prevention infrastructure and manuals (e.g., on social infrastructure construction, education, and training for medical personnel, and on situational response scenarios). The findings regarding this type corroborate the evidence in the past literature as more than service expansion was needed to address the gap in access to treatment and public health education after the COVID-19 pandemic [39]. It is necessary, therefore, to invest in social health centers and safety net hospitals in regions that provide expanded access to medical services and to establish an equitable treatment model for all patients to receive the treatment they need [39]. In Korea, public hospitals were at the center of treatment for COVID-19 patients, and the infrastructure of these nationally designated isolation and regional base hospitals was well utilized in responding to the pandemic. They indeed play an important role in all sorts of disaster situations as their transition to disaster-dedicated hospitals is easier (vs. that of private hospitals), and policy decisions according to national needs can be quickly transitioned into actions in such hospitals [40]. Therefore, the government needs to establish a systematic management system to provide a social prevention infrastructure, which should include both private hospitals and local communities. This can be carried out by establishing public hospitals in the case of a disaster. The government needs to establish public hospitals in the event of a disaster and establish a systematic management system for social prevention infrastructure that includes not only private hospitals, but also the local community. Nevertheless, social awareness of public hospitals is still low, and expectations for tertiary hospitals are high. Therefore, it seems necessary to spread positive awareness of public hospitals in preparation for the future infectious disease era. In addition, in the LTC hospitals, there are opinions that professional infection control education and systems are lacking, despite recent support for infection control personnel, so it seems necessary to provide specific education and training and establish manuals in connection with tertiary hospitals.

Upon comparing the different types that emerged in this study, it became clear that type I tertiary hospital nurses and type III LTC hospital nurses shared similarities regarding their focus on systems and manual construction. However, LTC hospital nurses emphasized the establishment of a one-step power and transport system, as well as social infrastructure. This may be because there are many situations in which an LTC hospital in Korea may need to transfer severely ill patients to a tertiary hospital. Meanwhile, the focus of type IV tertiary hospital nurses on emotional support was given relatively less importance in the LTC hospital setting. On the one hand, since tertiary hospital nurses mainly care for critically ill patients, it seems that they may have to deal with relatively high levels of stress; on the other hand, LTC hospital nurses tend to work two shifts and work overtime because of the lack of human power, which may explain why they gave more attention to compensation systems (type I in LTC hospital nurses) than emotional support. Therefore, when the government makes policies for tertiary hospital nurses, they should place greater emphasis on emotional support measures. In the context of LTC hospital nurses, policies could support them in the context of their immediate work, such as through providing small allowances, helping reduce the lack of protective equipment, facilities, and environments.

All nurses in our sample consensually highly agreed with Q9, namely on the need for an isolation ward or facility in the hospital to accommodate infected patients. This appears to be because isolation is important for patients with infectious diseases with high transmissibility. Among LTC hospital nurses, there was high agreement with Q12, which involves the establishment of a one-step power source and transfer system; this may be associated with the fact that seriously ill patients in LTC facilities must often be transferred to tertiary or infectious disease hospitals. These nurses also agreed with Q7, emphasizing the need to guarantee the maintenance and support for stable healthcare personnel at the national level. This agreement demonstrates the relative severity of nursing shortages in LTC hospitals, especially when compared with tertiary hospitals, and the situation where nurses must work two shifts because of human power shortage.

Among tertiary hospital nurses, there was a low level of agreement with Q17 on the need for emotional support from colleagues, and a low level of agreement among LTC hospital nurses with Q15, as it was seemingly difficult for them to deal with the lack of human power exclusively through their sense of duty. In summary, nurses from the two types of hospitals agreed that it was difficult to work without appropriate compensation and difficult to rely mostly on emotional support or a sense of calling for work. Therefore, we believe that both tertiary and LTC hospitals in Korea need realistic work support from the government to address the direct shortcomings that arise due to public health crises associated with an infectious disease. Our findings also demonstrate that “forced” job engagement without a relevant compensation system in place can have a negative impact on nurses’ perceptions of the healthcare safety net.

Upon comparing group characteristics, we observed that LTC hospital nurses tended to have a longer nursing experience and be older than tertiary hospital nurses. Meanwhile, type II tertiary hospital nurses showed the lowest average age in this group and were focused on realistic work support; type I LTC hospital nurses showed the lowest average age in this group and seemingly emphasized the compensation system and facility environmental support. Regarding the type with the highest average age in both groups, it was the type pertaining to government support in both tertiary and LTC hospital nurses. This goes to show that even for nurses within the same hospital environment, the perceptions about healthcare safety nets can differ by age. This sheds light on the potential of considering nurses’ age when developing healthcare policies, policies to improve hospital nurses’ work environment, and programs for tertiary or LTC hospitals.

This study has the following limitations. First, it has limited generalization because the sample comprises one tertiary hospital and one LTC hospital. Second, since most of the positive contents of the healthcare safety net were deemed necessary, it was difficult to clearly distinguish them because they overlap across types. Third, since the Q-methodology represents the participants’ subjectivity, it has limitations in categorically evaluating the results.

Despite these limitations, the perceptions of the healthcare safety net of nurses working in a tertiary hospital and in an LTC hospital were classified into four types, each through a Q-methodological approach. This study identified the differences in nurses’ perceptions by hospital setting, and that each hospital setting needs its own specific improvements. We hope that these data prove useful in improving the design of future measures to prevent the negative consequences of infectious disease outbreaks in these hospital settings.

## 7. Conclusions

This study identified and compared four perceptions of tertiary hospital nurses and four perceptions of LTC hospital nurses about healthcare safety nets. This study could be used as basic data for informing policy development and helping in the identification of deficiencies in the healthcare safety nets of the two hospital types. Dealing with these shortcomings may enable these hospitals to deliver high-quality nursing care for patients in an era characterized by infectious disease outbreaks.

We provide the following suggestions for future scholars. First, they could study systematic systems and policies to improve the healthcare safety nets according to the circumstances of different hospitals based on our findings. Second, we also suggest a tool to assess whether the healthcare safety net works well and its effectiveness.

## Figures and Tables

**Table 1:** Eigenvalue, Variance, Type Weights, and Characteristics of Tertiary Hospital Nurses (n = 32).

Type (n)	Eigenvalue	Variance (Cumulative)	ID	Gender	Age (yrs)	Marital Status	Educational Level	Total Clinical Career (yrs)	Tertiary Hospital Career (yrs)	Position	Adequate Care during the COVID-19 Pandemic	Difficulty System during the COVID-19 Pandemic	Type Weight	Factors Matrix					
														1	2	3	4	Com.	Pure.
1 (n = 10)	10.65	0.33 (0.33)	14	F	34	married	university	11.6	11.6	staff	no	hospital	2.62	0.83	0.06	0.14	0.11	0. 72	0.95
			28	F	31	married	university	8.0	8.0	staff	yes	individual	2.58	0.83	−0.26	0.22	0.08	0.80	0.85
			16	F	31	single	university	8.4	8.4	staff	yes	hospital	1.75	0.75	0.18	0.12	−0.16	0.64	0.89
			9	F	28	single	university	6.4	6.4	staff	yes	hospital	1.26	0.68	0.41	0.34	0.01	0.74	0.62
			18	F	31	single	master	9.5	9.5	staff	yes	social	1.15	0.66	−0.05	0.18	0.29	0.55	0.78
			22	F	29	single	university	7.0	6.4	staff	yes	hospital	1.01	0.62	0.28	0.29	0.01	0.54	0.71
			32	F	24	single	university	1.6	1.6	staff	yes	social	0.95	0.60	0.39	0.08	0.27	0.59	0.62
			31	F	25	single	university	3.1	3.1	staff	yes	hospital	0.73	0.53	0.46	0.33	−0.03	0.60	0.47
			25	F	31	single	university	6.0	6.0	staff	yes	hospital	0.67	0.50	0.35	0.28	0.16	0.48	0.53
			19	F	39	single	university	4.4	4.4	staff	yes	hospital	0.60	0.47	0.14	0.21	0.22	0.33	0.66
2 (n = 7)	2.58	0.08 (0.41)	30	F	28	single	university	5.5	5.5	staff	yes	hospital	2.11	0.29	0.79	0.09	−0.15	0.74	0.85
			20	F	36	married	master	11.8	11.8	staff	yes	hospital	1.65	0.04	0.74	0.24	−0.02	0.61	0.90
			29	F	24	single	university	1.3	1.3	staff	yes	hospital	1.20	0.05	0.67	0.06	0.24	0.51	0.88
			26	F	26	single	university	1.6	1.6	staff	no	hospital	1.04	0.29	0.63	0.15	0.38	0.65	0.61
			4	F	32	single	university	9.9	9.9	staff	no	social	0.90	0.09	0.59	0.39	0.11	0.52	0.67
			7	F	28	single	university	4.3	4.3	staff	yes	individual	0.54	0.05	0.44	−0.39	0.30	0.44	0.44
			17	F	28	married	university	5.7	5.7	staff	no	hospital	0.53	0.35	0.43	0.35	0.24	0.49	0.38
3 (n = 10)	2.29	0.07 (0.49)	15	F	42	married	university	19.3	19.3	staff	yes	hospital	2.69	0.11	0.16	0.83	0.12	0.74	0.93
			23	F	35	married	master	11.8	11.8	staff	no	hospital	1.87	0.28	0.16	0.77	0.23	0.75	0.79
			21	F	26	single	university	3.1	3.1	staff	yes	hospital	1.82	0.19	0.03	0.76	0.19	0.65	0.89
			8	F	29	single	university	6.7	6.7	staff	yes	no	1.44	0.35	0.00	0.71	0.07	0.63	0.80
			13	F	25	single	college	3.3	3.3	staff	yes	hospital	1.22	0.23	0.25	0.67	0.26	0.63	0.72
			10	F	31	married	university	7.6	7.6	staff	yes	social	0.97	0.18	0.30	0.61	0.03	0.50	0.75
			27	F	38	single	master	15	15	staff	yes	hospital	0.76	0.21	0.09	0.54	0.10	0.35	0.83
			1	F	43	married	master	13	13	staff	yes	hospital	0.61	0.13	0.23	0.47	0.37	0.43	0. 52
			12	F	30	single	university	6.8	6.8	staff	no	hospital	0.57	0.46	0.25	0.46	0.02	0.48	0.44
			6	F	30	single	university	3	3	staff	yes	hospital	0.54	0.27	0.29	0.44	0.43	0.54	0.36
4 (n = 5)	1.87	0.06 (0.54)	3	F	37	married	university	15	15	staff	yes	hospital	1.40	−0.06	0.08	0.23	0.71	0.56	0.89
			2	F	29	single	university	4.2	4.2	staff	yes	hospital	0.75	−0.10	−0.03	−0.16	0.54	0.32	0.89
			5	F	36	married	university	14	14	staff	yes	individual	0.70	0.27	0.03	−0.07	0.51	0.34	0.77
			24	F	29	single	university	5.3	5.3	staff	yes	hospital	0.58	0.12	0.31	0.21	0.46	0.36	0.58
			11	F	32	single	university	10	10	staff	yes	social	0.34	0.19	0.08	0.17	0.30	0.16	0.57

**Table 2:** Eigenvalue, Variance, Type Weights, and Characteristics of LTC Hospital Nurses (n = 33).

Type (n)	Eigenvalue	Variance (Cumulative)	ID	Gender	Age (yrs)	Marital status	Educational Level	Total Clinical Career (yrs)	LTC Hospital Career (yrs)	Position	Adequate Care during the COVID-19 Pandemic	Difficulty System during the COVID-19 Pandemic	Type Weight	Factors Matrix					
														1	2	3	4	Com.	Pure.
1 (n = 11)	13.52	0.41 (0.41)	27	M	30	single	college	2.5	2.5	staff	yes	hospital	1.87	0.77	0.14	0.16	0.31	0.73	0.81
			15	F	30	single	university	5.8	2.5	staff	yes	social	1.38	0.70	0.18	−0.04	0.20	0.57	0.87
			22	F	49	married	university	26	10.3	head	yes	hospital	1.17	0.66	0.33	0.09	0.36	0.68	0.64
			5	F	31	single	university	9.6	8.5	staff	yes	hospital	1.15	0.66	0.11	0.17	0.39	0.63	0.69
			10	F	50	single	university	15	8.3	staff	yes	hospital	1.12	0.65	0.54	0.16	0.19	0.77	0.55
			24	M	27	single	university	1.8	1.8	staff	yes	hospital	1.05	0.63	0.38	0.27	0.13	0.63	0.63
			9	F	52	married	university	29	9	head	yes	hospital	1.00	0.62	0.35	0.00	0.13	0.52	0.73
			4	F	34	single	university	11	10.8	staff	yes	social	0.82	0.56	0.42	−0.08	−0.06	0.50	0.63
			1	F	49	married	master	3	2.2	staff	yes	social	0.79	0.55	0.04	0.44	0.26	0.57	0.53
			14	F	32	married	university	3	0.3	staff	yes	social	0.76	0.54	0.29	0.24	0.45	0.64	0.46
			31	F	31	single	college	8	8	staff	yes	hospital	0.55	0.44	0.39	0.09	0.20	0.39	0.49
2 (n = 10)	1.88	0.06 (0.47)	26	F	56	married	university	4.9	4.9	staff	yes	hospital	1.57	0.04	0.73	0.00	0.33	0.65	0.83
			16	F	55	married	university	13	3	staff	yes	hospital	1.15	0.29	0.66	−0.70	0.10	0.53	0.81
			32	F	51	married	university	15	11	staff	yes	hospital	1.07	0.09	0.64	0.34	0.24	0.59	0.69
			30	F	39	married	university	14.8	6.8	staff	no	hospital	1.07	0.24	0.64	0.43	0.22	0.70	0.58
			13	F	48	married	university	26	8	head	yes	hospital	0.86	0.26	0.58	0.23	0.25	0.51	0.65
			29	F	45	married	college	20	4	staff	yes	hospital	0.82	0.43	0.56	0.32	0.23	0.66	0.48
			21	M	28	single	university	1.2	0.8	staff	no	hospital	0.79	0.30	0.55	0.04	0.38	0.53	0.57
			8	F	49	single	university	20	10	staff	yes	hospital	0.76	0.24	0.54	−0.11	0.40	0.51	0.57
			25	F	51	married	university	27	10.5	head	no	hospital	0.69	0.39	0.51	0.29	0.14	0.51	0.51
			28	F	55	married	university	1.7	1.7	staff	yes	hospital	0.41	0.11	0.36	−0.03	0.03	0.15	0.90
3 (n = 4)	1.53	0.05 (0.51)	12	F	62	married	university	20	8.8	staff	yes	social	1.03	0.33	−0.02	0.63	0.33	0.61	0.64
			7	F	48	married	master	15.8	7.7	head	yes	hospital	1.01	0.25	0.39	0.62	0.04	0.60	0.64
			19	F	45	married	university	20	10	staff	yes	hospital	0.56	0.23	0.42	0.45	0.02	0.43	0.46
			2	F	52	married	master	28	12	head	no	hospital	0.43	−0.14	−0.08	0.37	0.03	0.16	0.84
4 (n = 8)	1.36	0.04 (0.55)	33	F	55	married	college	18	14	head	yes	hospital	1.30	0.14	0.21	0.08	0.69	0.54	0.87
			17	F	54	married	college	20	7.8	staff	no	hospital	1.28	0.29	0.03	0.30	0.68	0.64	0.73
			6	F	51	married	university	9.8	4	head	yes	hospital	1.03	0.31	0.41	0.22	0.63	0.70	0.56
			18	F	59	married	university	20	1.7	staff	yes	hospital	0.86	0.23	0.57	0.01	0.58	0.71	0.47
			20	F	59	married	master	20	11	staff	yes	hospital	0.64	0.35	0.31	0.42	0.49	0.63	0.38
			3	F	53	married	college	20	15	staff	yes	social	0.63	0.29	−0.01	0.18	0.48	0.35	0.66
			23	F	48	single	university	15	12	staff	yes	hospital	0.60	0.30	0.47	0.31	0.47	0.63	0.35
			11	F	59	married	college	13.8	8.7	staff	yes	hospital	0.57	0.07	0.29	−0.15	0.45	0.32	0.64

**Table 3:** Q-Statements and Z-Scores of Perceptions Toward Healthcare Safety Nets in Tertiary Hospital Nurses (n = 32).

No	Q-Statements	Z-Score			
		Type I (n = 10)	Type II (n = 7)	Type III (n = 10)	Type IV (n = 15)
Q1	I think access to healthcare services should be guaranteed, such as using and receiving support from selected clinics.	1.1	−0.4	1.4	0.0
Q2	I believe that disaster healthcare response, infectious disease prevention and management, and health education should be provided at the national and local government levels.	1.3	−0.3	1.7	0.2
Q3	I think that psychological counseling should be provided to reduce the anxiety, stress, and insomnia of medical personnel.	−1.2	0.1	1.0	0.7
Q4	I think it is necessary to ensure measures to protect from infection and for early detection, such as testing, self-isolation, hand washing, and wearing a mask.	1.5	0.5	1.5	−0.7
Q5	I think it is necessary to establish and activate clinical laboratories and research systems to create scientific evidence.	1.0	−0.6	0.7	0.1
Q6	I think there is a need for education and training programs for healthcare personnel and related personnel.	1.1	0.0	1.0	0.8
Q7	I believe that maintenance and support measures for stable healthcare personnel should be guaranteed at the national level.	1.0	−0.1	1.6	1.3
Q8	I think that an appropriate compensation system for overtime work should be established in the form of monetary compensation, vacations, awards, among others.	0.7	2.0	2.0	1.4
Q9	I think there is a need for an isolation ward or facility where patients can be immediately isolated in the event of an outbreak.	0.3	−0.0	0.4	0.9
Q10	I think it is needs to secure the number of nurses when nursing patients with infectious diseases.	0.5	2.0	0.8	0.2
Q11	I think it is needs to secure infectious disease items such as hand washing products, masks, protective gear, and gloves.	1.5	1.8	0.7	−0.3
Q12	I think that a one-step power source and transfer system is needed in the event of infected patients.	−0.0	0.8	−0.2	1.0
Q13	I think that high-risk facilities should be able to handle confirmed cases well.	0.5	0.9	−0.2	−0.0
Q14	I think we need a mature national consciousness that favors the common interest a little more than the individual.	−0.5	−0.9	−0.3	−1.7
Q15	I think that even if I must work in a second shift because of a lack of human power, I can endure it because I have a sense of calling for the job.	−2.2	−2.3	−2.4	−0.6
Q16	I think it is necessary to establish an emotional nursing support system so that patients do not feel anxious about contracting COVID-19.	−1.1	−0.9	−1.1	0.8
Q17	I believe that people who are not confirmed to have COVID-19 need emotional support from colleagues so that they can withstand the shifts.	−1.2	−0.6	−0.2	−0.7
Q18	I think that people other than patients and medical staff need psychiatric treatment and counseling programs.	−1.6	−1.0	−0.2	1.2
Q19	When nursing patients with infectious diseases, I think that information sharing among healthcare personnel should be well done.	0.1	1.1	0.1	1.3
Q20	I think we need a rest area where we can take a break while wearing protective gear.	−1.3	0.9	0.3	0.4
Q21	I think it is necessary to establish healthcare treatment systems for suspected or infected patients through non-face-to-face methods.	0.4	−0.8	−0.6	−0.3
Q22	I think it is necessary to secure sufficient transportation, such as helicopters and ambulances, in case of an emergency.	−0.5	0.9	−0.4	−1.8
Q23	We believe in the provision of necessary care to patients regardless of financial situation, coverage of insurance, medically underserved area, or class.	0.0	−1.4	−0.4	−0.1
Q24	I think it is necessary to have efforts to invest in equity to ensure a safe and fair social and digital infrastructure.	−0.5	1.7	−0.6	−1.0
Q25	I believe that the essential role of public health is to provide essential healthcare services that reflect community issues and needs.	0.2	−0.4	−0.9	−0.7
Q26	I think it is necessary to rearrange the government organization that can operate a comprehensive and effective crisis communication system and a quarantine system.	−0.5	−0.1	−0.6	−0.3
Q27	I think social distancing or maintaining an effective system is necessary.	−0.0	0.1	−1.8	−1.3
Q28	I think the infection support team should be able to provide quick support to nurses, just like the CPR team.	0.3	−0.1	−1.2	2.2
Q29	I think it is necessary to prepare a manual for actions and situational response scenarios in the event of a COVID-19 patient.	1.6	0.7	−0.2	1.1
Q30	I think it is necessary to publicize and announce immediate needs for prevention measures against infectious diseases at each stage of the public health crisis.	0.7	0.2	−0.4	−1.2
Q31	I think that staff and patients are safely protected from infectious diseases.	−0.4	0.5	−0.7	−0.8
Q32	I think it is necessary to protect personal information of the first infected person.	−1.2	−1.4	0.0	−1.8
Q33	There are cases where the patient punches or swears, and in these cases, I think it is necessary to have a security guard.	−1.4	0.6	−0.7	−0.4

**Table 4:** Q-Statements and Z-Scores of Perceptions Toward Healthcare Safety Nets in LTC Hospital Nurses (n = 33).

No	Q-Statements	Z-Score			
		Type I (n = 11)	Type II (n = 10)	Type III (n = 4)	Type IV (n = 8)
Q1	I think access to healthcare services should be guaranteed, such as using and receiving support from selected clinics.	−0.2	−0.7	0.5	1.6
Q2	I believe that disaster healthcare response, infectious disease prevention and management, and health education should be provided at the national and local government levels.	−0.2	0.0	−0.1	.8
Q3	I think that psychological counseling should be provided to reduce the anxiety, stress, and insomnia of medical personnel.	−1.6	0.8	−0.4	0.1
Q4	I think it is necessary to ensure measures to protect from infection and for early detection, such as testing, self-isolation, hand washing, and wearing a mask.	2.0	0.1	0.5	1.7
Q5	I think it is necessary to establish and activate clinical laboratories and research systems to create scientific evidence.	−0.8	−1.1	0.3	−0.5
Q6	I think there is a need for education and training programs for healthcare personnel and related personnel.	0.2	−0.4	1.0	−0.6
Q7	I believe that maintenance and support measures for stable healthcare personnel should be guaranteed at the national level.	1.4	0.7	1.4	1.4
Q8	I think that an appropriate compensation system for overtime work should be established in the form of monetary compensation, vacations, awards, among others.	2.3	2.0	0.5	1.8
Q9	I think there is a need for an isolation ward or facility where patients can be immediately isolated in the event of an outbreak.	1.4	1.7	2.1	1.6
Q10	I think it is needs to secure the number of nurses when nursing patients with infectious diseases.	1.3	2.0	−0.3	1.8
Q11	I think it is needs to secure infectious disease items such as hand washing products, masks, protective gear, and gloves.	1.2	.2	1.6	.3
Q12	I think that a one-step power source and transfer system is needed in the event of infected patients.	0.8	1.6	1.6	1.3
Q13	I think that high-risk facilities should be able to handle confirmed cases well.	0.7	0.7	0.7	0.7
Q14	I think we need a mature national consciousness that favors the common interest a little more than the individual.	−0.8	−0.5	0.0	−1.0
Q15	I think that even if I must work in a second shift because of a lack of human power, I can endure it because I have a sense of calling for the job.	−1.7	−1.9	−1.8	−1.2
Q16	I think it is necessary to establish an emotional nursing support system so that patients do not feel anxious about contracting COVID-19.	−1.0	−0.6	−0.3	−0.8
Q17	I believe that people who are not confirmed to have COVID-19 need emotional support from colleagues so that they can withstand the shifts.	−0.9	−0.9	−0.2	0.1
Q18	I think that people other than patients and medical staff need psychiatric treatment and counseling programs.	−1.2	−0.6	−0.9	−0.3
Q19	When nursing patients with infectious diseases, I think that information sharing among healthcare personnel should be well done.	0.2	0.3	0.2	−0.1
Q20	I think we need a rest area where we can take a break while wearing protective gear.	1.0	−0.1	−1.8	−0.5
Q21	I think it is necessary to establish healthcare treatment systems for suspected or infected patients through non-face-to-face methods.	−1.2	−0.2	−0.5	−0.1
Q22	I think it is necessary to secure sufficient transportation, such as helicopters and ambulances, in case of an emergency.	0.1	−0.2	−1.5	−0.4
Q23	We believe in the provision of necessary care to patients regardless of financial situation, coverage of insurance, medically underserved area, or class.	−0.5	−1.3	−0.3	−0.9
Q24	I think it is necessary to have efforts to invest in equity to ensure a safe and fair social and digital infrastructure.	−1.0	−1.3	−0.1	−1.9
Q25	﻿I believe that the essential role of public health is to provide essential healthcare services that reflect community issues and needs.	−0.6	−1.2	−0.1	−1.2
Q26	I think it is necessary to rearrange the government organization that can operate a comprehensive and effective crisis communication system and a quarantine system.	−0.1	0.3	0.4	−0.4
Q27	I think social distancing or maintaining a positive system is necessary.	−0.2	−0.8	−0.6	−0.8
Q28	I think the infection support team should be able to provide quick support to nurses, just like the CPR team.	0.3	1.7	0.5	−0.9
Q29	I think it is necessary to prepare a manual for actions and situational response scenarios in the event of a COVID-19 patient.	0.4	0.8	1.4	0.4
Q30	I think it is necessary to publicize and announce the immediate need for prevention measures against infectious diseases at each stage of the public health crisis.	−0.1	−0.9	0.2	−0.9
Q31	I think that staff and patients are safely protected from infectious diseases.	0.5	0.5	−0.5	−0.2
Q32	I think it is necessary to protect the personal information of the first infected person.	−0.5	−0.9	−1.4	−1.2
Q33	There are cases where the patient punches or swears, and in these cases, I think it is necessary to have a security guard.	−1.1	−0.0	−2.0	−0.1

## Data Availability

Not applicable.

## Appendix A. Q-Statements with Positive and Negative Differences in Z-Score for Each Type and Consensus Views in Tertiary Hospital Nurses (n = 32)

**Type**	**No**	**Q-Statements**	**Z-Score**	**Average**	**Difference**
I	Q30	I think it is necessary to publicize and announce the immediate need for prevention measures against infectious diseases at each stage of the public health crisis.	0.68	−0.49	1.17
	Q29	I think it is necessary to prepare a manual for actions and situational response scenarios in the event of a COVID-19 patient.	1.64	0.52	1.11
	Q4	I think it is necessary to ensure measures to protect from infection and for early detection, such as testing, self-isolation, hand washing, and wearing a mask.	1.51	0.44	1.06
	Q21	I think it is necessary to establish healthcare treatment systems for suspected or infected patients through non-face-to-face methods.	0.44	−0.57	1.009
	Q8	I think that an appropriate compensation system for overtime work should be established in the form of monetary compensation, vacations, awards, among others.	0.67	1.78	−1.12
	Q33	There are cases where the patient punches or swears, and in these cases, I think it is necessary to have a security guard.	−1.44	−0.18	−1.26
	Q18	I think that people other than patients and medical staff need psychiatric treatment and counseling programs.	−1.58	0.02	−1.60
	Q3	I think that psychological counseling should be provided to reduce the anxiety, stress, and insomnia of medical personnel.	−1.23	0.59	−1.82
	Q20	I think we need a rest area where we can take a break while wearing protective gear.	−1.32	0.55	−1.86
II	Q22	I think it is necessary to secure sufficient transportation, such as helicopters and ambulances, in case of an emergency.	0.91	−0.88	1.79
	Q10	I think it is needs to secure the number of nurses when nursing patients with infectious diseases.	1.96	0.51	1.45
	Q33	There are cases where the patient punches or swears, and in these cases, I think it is necessary to have a security guard.	0.58	−0.85	1.43
	Q11	I think it is needs to secure infectious disease items such as hand washing products, masks, protective gear, and gloves.	1.80	0.61	1.18
	Q27	I think social distancing or maintaining a positive system is necessary.	0.068	−1.04	1.10
	Q31	I think that staff and patients are safely protected from infectious diseases.	0.50	−0.59	1.09
	Q20	I think we need a rest area where we can take a break while wearing protective gear.	0.88	−0.19	1.07
	Q24	I think it is necessary to have efforts to invest in equity to ensure a safe and fair social and digital infrastructure.	−1.73	−0.71	−1.03
	Q5	I think it is necessary to establish and activate clinical laboratories and research systems to create scientific evidence.	−0.59	0.60	−1.18
	Q1	I think access to healthcare services should be guaranteed, such as using and receiving support from selected clinics.	−0.36	0.86	−1.22
	Q23	We believe in the provision of necessary care to patients regardless of financial situation, coverage of insurance, medically underserved area, or class.	−1.44	−0.17	−1.27
	Q2	I believe that disaster healthcare response, infectious disease prevention and management, and health education should be provided at the national and local government levels.	−0.28	1.06	−1.34
	Q7	I believe that maintenance and support measures for stable healthcare personnel should be guaranteed at the national level.	−0.12	1.29	−1.41
III	Q32	I think it is necessary to protect personal information when it becomes the first infected person.	0.02	−1.49	1.51
	Q2	I believe that disaster healthcare response, infectious disease prevention and management, and health education should be provided at the national and local government levels.	1.74	0.39	1.36
	Q3	I think that psychological counseling should be provided to reduce the anxiety, stress, and insomnia of medical personnel.	1.01	−0.15	1.16
	Q1	I think access to healthcare services should be guaranteed, such as using and receiving support from selected clinics.	1.39	0.27	1.12
	Q27	I think social distancing or maintaining a positive system is necessary.	−1.77	−0.42	−1.35
	Q29	I think it is necessary to prepare a manual for actions and situational response scenarios in the event of a COVID-19 patient.	−0.25	1.15	−1.40
	Q28	I think the infection support team should be able to provide quick support to nurses, just like the CPR team.	−1.23	0.80	−2.03
IV	Q28	I think the infection support team should be able to provide quick support to nurses, just like the CPR team.	2.22	−0.36	2.58
	Q18	I think that people other than patients and medical staff need psychiatric treatment and counseling programs.	1.25	−0.92	2.17
	Q16	I think it is necessary to establish an emotional nursing support system so that patients do not feel anxious about contracting COVID-19.	0.77	−1.04	1.82
	Q15	I think that even if I must work in a second shift because of a lack of human power, I can endure it because I have a sense of calling for the job.	−0.55	−2.31	1.76
	Q14	I think we need a mature national consciousness that favors the common interest a little more than the individual.	−1.67	−0.53	−1.14
	Q30	I think it is necessary to publicize and announce the immediate need for prevention measures against infectious diseases at each stage of the public health crisis.	−1.25	0.16	−1.40
	Q11	I think it is needs to secure infectious disease items such as hand washing products, masks, protective gear, and gloves.	−0.29	1.31	−1.60
	Q21	I think it is necessary to establish healthcare treatment systems for suspected or infected patients through non-face-to-face methods.	−1.76	0.01	−1.77
	Q4	I think it is necessary to ensure measures to protect from infection and for early detection, such as testing, self-isolation, hand washing, and wearing a mask.	−0.66	1.17	−1.83

## Appendix B. Consensus Items and Average Z-Score in Tertiary Hospital Nurses (n = 32)

**Type**	**No**	**Q-Statements**	**Z-Score**
Consensus Items	Q9	I think there is a need for an isolation ward or facility where patients can be immediately isolated in the event of an outbreak.	0.38
	Q26	I think it is necessary to rearrange the government organization that can operate a comprehensive and effective crisis communication system and a quarantine system.	−0.39
	Q17	I believe that people who are not confirmed to have COVID-19 need emotional support from colleagues so that they can withstand the shifts.	−0.67

## Appendix C. Q-Statements with Positive and Negative Differences in Z-Score for Each Type and Consensus Views in LTC Hospital Nurses (n = 33)

**Type**	**No**	**Q-Statements**	**Z-Score**	**Average**	**Difference**
I	Q20	I think we need a rest area where we can take a break while wearing protective gear.	1.01	−0.80	1.81
	Q4	I think it is necessary to ensure measures to protect from infection and for early detection, such as testing, self-isolation, hand washing, and wearing a mask.	1.96	0.77	1.20
	Q8	I think that an appropriate compensation system for overtime work should be established in the form of monetary compensation, vacations, awards, among others.	2.27	1.42	0.84
	Q12	I think that a one-step power source and transfer system is needed in the event of infected patients.	0.78	1.49	−0.72
	Q21	I think it is necessary to establish healthcare treatment systems for suspected or infected patients through non-face-to-face methods.	−1.19	−0.24	−0.95
	Q3	I think that psychological counseling should be provided to reduce the anxiety, stress, and insomnia of medical personnel.	−1.62	0.17	−1.79
II	Q28	I think the infection support team should be able to provide quick support to nurses, just like the CPR team.	1.70	−0.06	1.76
	Q3	I think that psychological counseling should be provided to reduce the anxiety, stress, and insomnia of medical personnel.	0.79	−0.64	1.42
	Q33	There are cases where the patient punches or swears, and in these cases, I think it is necessary to have a security guard.	−0.01	−1.06	1.05
	Q10	I think it is needs to secure the number of nurses when nursing patients with infectious diseases.	1.97	0.93	1.04
	Q11	I think it is needs to secure infectious disease items such as hand washing products, masks, protective gear, and gloves.	0.17	1.05	−0.88
	Q4	I think it is necessary to ensure measures to protect from infection and for early detection, such as testing, self-isolation, hand washing, and wearing a mask.	0.15	1.37	−1.22
	Q1	I think access to healthcare services should be guaranteed, such as using and receiving support from selected clinics.	−0.67	0.64	−1.31
III	Q24	I think it is necessary to have efforts to invest in equity to ensure a safe and fair social and digital infrastructure.	−0.11	−1.39	1.28
	Q6	I think there is a need for education and training programs for healthcare personnel and related personnel.	1.00	−0.26	1.26
	Q5	I think it is necessary to establish and activate clinical laboratories and research systems to create scientific evidence.	0.34	−0.78	1.11
	Q11	I think it is needs to secure infectious disease items such as hand washing products, masks, protective gear, and gloves.	1.59	0.58	1.01
	Q22	I think it is necessary to secure sufficient transportation, such as helicopters and ambulances, in case of an emergency.	−1.53	−0.21	−1.32
	Q8	I think that an appropriate compensation system for overtime work should be established in the form of monetary compensation, vacations, awards, among others.	0.50	2.01	−1.51
	Q33	There are cases where the patient punches or swears, and in these cases, I think it is necessary to have a security guard.	−1.99	−0.40	−1.59
	Q20	I think we need a rest area where we can take a break while wearing protective gear.	−1.83	0.14	−1.97
	Q10	I think it is needs to secure the number of nurses when nursing patients with infectious diseases.	−0.31	1.69	−2.00
IV	Q1	I think access to healthcare services should be guaranteed, such as using and receiving support from selected clinics.	1.56	−0.10	1.67
	Q2	I believe that disaster healthcare response, infectious disease prevention and management, and health education should be provided at the national and local government levels.	0.85	−0.11	0.96
	Q7	I believe that maintenance and support measures for stable healthcare personnel should be guaranteed at the national level.	0.13	−0.67	0.79
	Q6	I think there is a need for education and training programs for healthcare personnel and related personnel.	−0.57	0.26	−0.84
	Q24	I think it is necessary to have efforts to invest in equity to ensure a safe and fair social and digital infrastructure.	−1.91	−0.80	−1.12
	Q28	I think the infection support team should be able to provide quick support to nurses, just like the CPR team.	−0.93	0.82	−1.75

## Appendix D. Consensus Items and Average Z-Score in LTC Nurses (n = 33)

**Type**	**No**	**Q-Statements**	**Z-Score**
Consensus Items	Q9	I think there is a need for an isolation ward or facility where patients can be immediately isolated in the event of an outbreak.	1.67
	Q12	I think that a one-step power source and transfer system is needed in the event of infected patients.	1.31
	Q7	I believe that maintenance and support measures for stable healthcare personnel should be guaranteed at the national level.	1.23
	Q15	I think that even if I must work in a second shift because of a lack of human power, I can endure it because I have a sense of calling for the job.	−1.63
